# Thermal and Biomechanical Responses of Amateur, Elite and World Cup Athletes During a World Cup Sprint Triathlon in the Heat

**DOI:** 10.1007/s40279-025-02193-7

**Published:** 2025-03-27

**Authors:** Carl James, Borja Muniz-Pardos, Mohammed Ihsan, Ka-Kay Lo, Wing-Kai Lam, Dani Peña Iglesias, Konstantinos Angeloudis, Yi Teng, Jiao Jiao, Ke Hu, KaKi Wong, Fergus Guppy, Sébastien Racinais, Samuel Chalmers, Sergio Migliorini, Kenneth Wu, Yannis Pitsiladis

**Affiliations:** 1 Department of Sports and Health Science, Academy of Wellness and Human Development, Faculty of Arts and Social Sciences, Hong Kong Baptist University, Kowloon Tong, Hong Kong, China; 2 EXER-GENUD (Growth, Exercise, NUtrition and Development) Research Group, FIMS Collaborating Center of Sports Medicine, University of Zaragoza, Saragossa, Spain; 3 Scientific Conditioning Centre, Elite Training Science and Technology Division, Hong Kong Sports Institute, Hong Kong, China; 4 Human Telemetrics LTD, London, England; 5 Institute of Life and Earth Sciences, School of Energy Geosciences Infrastructure and Society, Heriot Watt University, Edinburgh, UK; 6 Department of Computing Science, Guangdong University of Education, Guangzhou, China; 7 Environmental Stress Unit, CREPS Montpellier Font-Romeu, Montpellier, France; 8 UMR 866 INRAE/University of Montpellier, DMEM, Montpellier, France; 9 Alliance for Research in Exercise, Nutrition and Activity (ARENA), Allied Health and Human Performance, University of South Australia, Adelaide, Australia; 10 World Triathlon Medical Committee, World Triathlon, Lausanne, Switzerland; 11 Hong Kong Triathlon (Event Chief Medical Officer), Hong Kong, China

## Abstract

**Objectives:**

Core temperature (T_CORE_), skin temperature (T_TORSO_) and running kinematics were measured across different athlete categories at a World Cup Sprint Triathlon, occurring during a heatwave (~ 25–31 °C Wet Bulb Globe Temperature [WBGT]).

**Methods:**

Sixty-six triathletes participated: 21 World Cup (7 females), 32 Hong Kong-Elite (HK-Elite; 8 females) and 13 Amateur (6 females).

**Results:**

Seventeen triathletes displayed a T_CORE_ > 40.0 °C and two > 41.0 °C. Peak T_CORE_ was not different between athlete categories (World Cup: 39.7 ± 0.6 °C; HK-Elite: 39.9 ± 0.8 °C; Amateur: 39.5 ± 0.8 °C; *p* = 0.357). However, there was an interaction between race phase and category (*p* = 0.001). Changes in T_CORE_ for World Cup (2.4 ± 0.4 °C) and HK-Elite (2.5 ± 1.0 °C) were greater than for Amateurs (1.5 ± 0.7 °C). Peak T_TORSO_ was higher in HK-Elites during afternoon races compared with morning World Cup races (*p* < 0.001). T_TORSO_ reduced during the swim (*p*_bonf_ < 0.001), then increased during the bike (*p*_bonf_ < 0.001) but not run (*p*_bonf_ = 1.00). World Cup athletes (3.15 ± 0.23 m) displayed longer strides (HK-Elites: 2.64 ± 0.35 m; Amateurs: 2.18 ± 0.30 m; *p*_bonf_ < 0.001), shorter contact times (209.3 ± 13.7 ms; HK-Elites: 237.8 ± 23.0 ms; Amateurs: 262.9 ± 31.0 ms, *p*_bonf_ < 0.001) and higher stride frequency (182.9 ± 6.3 strides.min^−1^) than HK-Elites (173.9 ± 6.8 strides.min^−1^) and Amateurs (173.2 ± 8.7 strides.min^−1^, *p*_bonf_ < 0.001), which were comparable. There were no biomechanical changes over time and no interactions.

**Conclusion:**

Different athlete categories displayed comparable peak T_CORE_ responses. Amateur triathletes tolerated T_CORE_ > 40.0 °C without heat illness symptoms. T_CORE_ may rise > 41 °C during a sprint triathlon held under *Blue* flag conditions (~ 26 °C WBGT), questioning the suitability of sprint-distance triathlons as a safer alternative to Olympic-distance triathlons under *Red/Black* flag conditions (> 30.1 °C WBGT).

## Key Points

**Table:** 

Mitigating the risks of exertional heat illness remains a major challenge for event organisers of large sporting competitions, which often include amateur through to professional participants. How athletes of different training status respond at the same event is unclear.
The presentation of core temperatures > 40 °C appears to be independent of athlete level. Extreme body temperatures (2 individuals > 41 °C) occurred during a sprint triathlon, which is a stated modification purported to lower risk for Olympic-distance triathlon.
Reducing triathlon events to a sprint distance may not sufficiently reduce the risk of heat illness, earlier start times may be a more suitable strategy.

## Introduction

There is growing interest in monitoring the performance, physiology and health of individuals during sporting competition [1], and the International Olympic Committee (IOC) has acknowledged a potential role for live monitoring technologies to protect health during endurance competitions in hot weather [2]. Monitoring outdoor competitions aids understanding of the development and etiology of exertional heat illnesses (EHI), without inherent limitations of laboratory trials such as exercise specificity, competition motivation and ethical precautions imposing exercise cessation (i.e. upon attaining a core temperature (T_CORE_) of 39.5–40 °C) [3]. Notably, a growing body of literature demonstrates that T_CORE_ in elite athletes routinely exceeds these safety limits during competition [4–9]. Currently, EHI risk management at many international competitions is based upon predicted or measured environmental heat stress [10]. Some international sporting federations, including World Triathlon, recommend rescheduling, cancelling, or shortening competitions under specific weather scenarios (e.g. reduce Olympic distance to sprint event when wet bulb globe temperature [WBGT] is > 30 °C) [11]. However, supplementing such strategies with monitoring of the heat strain individuals are experiencing, rather than utilising environmental conditions in isolation, could more precisely characterise EHI risks.

Recent literature advocates integrating multiple sensors to understand EHI development, by combining thermoregulatory metrics with performance and biomechanical parameters such as pace and gait variability [9, 12, 13]. In particular, ataxic gait may help to predict the most serious EHI, exertional heatstroke (EHS) [12]. This reflects the clinical diagnosis of EHS being a T_CORE_ > 40 °C, alongside central nervous system dysfunction [14]. As well as the technical challenges of developing multi-sensor technologies to monitor large cohorts in real time [15], effective solutions also require considerable normative data to contextualise human responses during different sporting events. However, there is a dearth of competition data on elite athletes in general [16], especially relating to T_CORE_ responses whilst competing in the heat, as has been highlighted within expert commentaries [17] and by the IOC [2, 6]. Furthermore, to our knowledge, the biomechanical responses of athletes during multi-modal sports such as triathlon, where individuals enter the run phase with an elevated body temperature and partially fatigued, have yet to be reported.

Sporting federations also often combine high-profile international events with amateur races on the same day, such as the public and Olympic marathons at Paris 2024. In these scenarios, world-class and amateur athletes compete under similar environmental conditions with potentially disparate thermal and biomechanical responses [18]. Understanding the variability of individual responses and modifying effects of factors such as age, sex and fitness level is necessary for interpreting data and creating predictive models or early-warning systems that could use live monitoring technologies to support medical personnel in pre-empting EHI in athletes. Moreover, competition data can help shape sport-specific EHI policies of international sporting federations and the IOC for hot weather competitions [2] for all participating categories, which is pertinent as amateur categories tend to have the largest participation. Therefore, investigating the responses of different standards of athletes is a priority area across many sports. In triathlon, no data are available documenting the thermal responses of different standards of athletes at the same event. Given environmental conditions remain a recognised risk factor within short-course triathlon, a need has been highlighted to investigate the responses of large numbers of athletes [19]. This is especially pertinent given reducing race distance is a stated EHI prevention policy for triathlon and other endurance sports; however, the thermal responses of elite athletes to shorter, ‘sprint’ triathlons are unknown.

The aims of this study were to characterise the T_CORE_, skin temperature (T_TORSO_) and running kinematic responses of different athlete categories within a Triathlon World Cup sprint race, to support policymaking that can enhance athlete safety in hot conditions.

## Methods

### Design

**Fig. 1:**
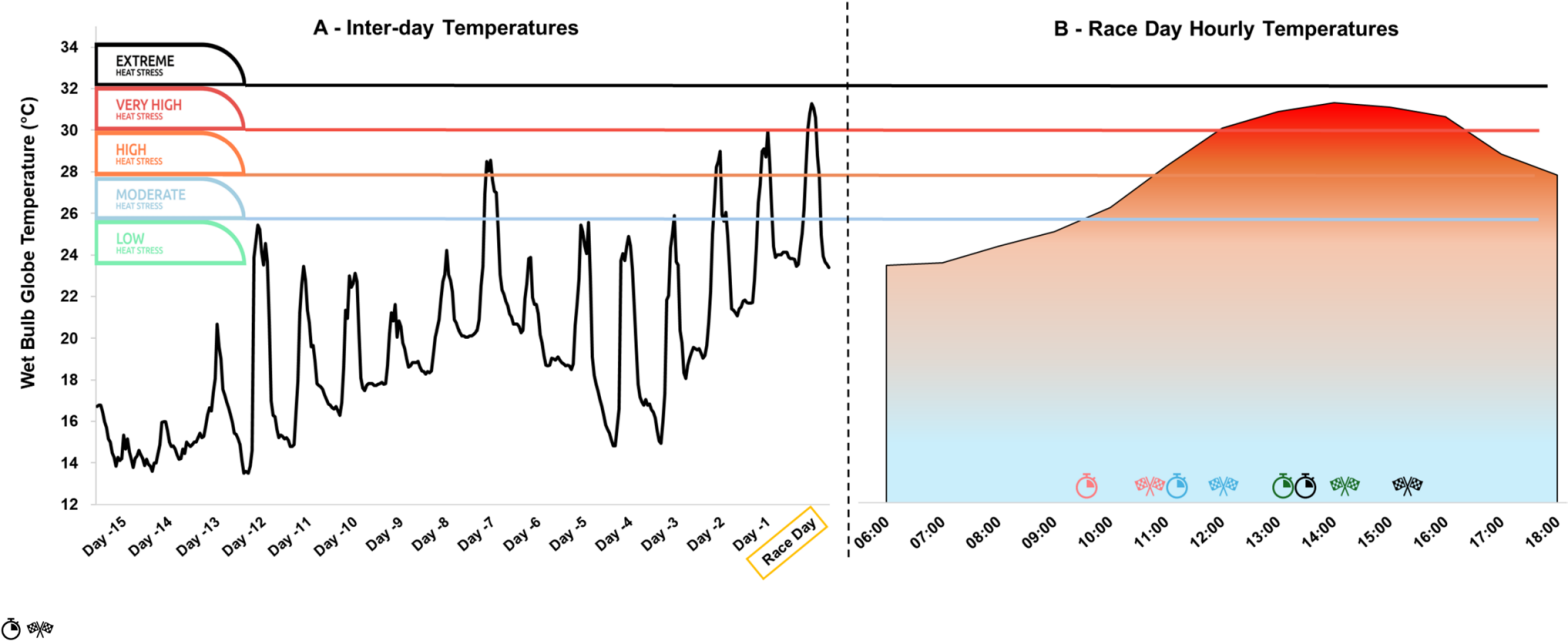
Environmental conditions **A** across two weeks prior to international sprint triathlon and **B** on race day.  denotes race start.  denotes race finish. World Cup female (start 09:30) = red symbols, World Cup male (start 11:00) = blue symbols, Hong Kong Elite male and female (start 13:00 and 13:03 respectively) = green symbols, all amateur categories (start 13:30–14:00) = black symbols. Coloured flags align with World Triathlon Heat Stress guidelines [11], whereby low heat stress = *Green* flag (< 25.7 °C wet bulb globe temperature [WBGT]), moderate heat stress = *Blue* flag (25.7–27.8 °C WBGT), high heat stress = *Orange* flag (27.9–30.0 °C WBGT), very high heat stress = *Red* flag (30.1–32.2 °C WBGT) and extreme heat stress = *Black* flag (> 32.2 °C WBGT)

A quasi-experimental approach was used to investigate the thermal and biomechanical responses of triathletes of different performance standards. Data collection took place during an international sprint triathlon on 24th March 2024, in Hong Kong, China. Race distances were 750-m swim, 20-km cycle and 5-km run. Across the day, 538 athletes participated in different categories, from 9.30 a.m. until 3 p.m. (Fig. 1).

### Participants

Sixty-six triathletes volunteered for the study. This included 21 World Cup, 32 Hong Kong Elite (HK-Elite) and 13 Amateur (‘age-group’) triathletes (Table 1). HK-Elite comprised predominantly junior athletes within the regional high-performance programme, whilst World Cup and Amateur groups consisted of senior athletes with a wide range of ages. The protocol complied with the Declaration of Helsinki and was approved by the local Research Ethics Committee (REC/23–24/0028). Written informed consent was obtained before the race from participants as well as from parents or guardians for participants under 18 years of age.

**Table 1:** Breakdown of race categories and athlete characteristics

Race category	Sex	*n*	Perf. tier	Tier %	Age (years)	Stature (cm)	Mass (kg)
World cup	Female	7	4/5	29%/71%	29 ± 6	167 ± 7	56.5 ± 5.2
	Male	14	4/5	79%/21%	26 ± 4	179 ± 7	70.6 ± 5.6
HK-Elite	Female	8	3/4	75%/25%	20 ± 5	166 ± 6	55.4 ± 4.0
	Male	24	3/4	83%/17%	18 ± 2	173 ± 7	61.1 ± 8.6
Amateur/age group	Female	6	1/2	83%/17%	38 ± 6	169 ± 4	62.2 ± 6.5
	Male	7	1/2	57%/43%	38 ± 8	175 ± 9	77.5 ± 9.1
Overall		66			25 ± 9	173 ± 8	63.5 ± 9.6

‘Perf. Tier’ represents performance classification in accordance with McKay et al. [20], whereby 0 = sedentary, 1 = recreationally active, 2 = trained/developmental, 3 = highly trained/national level, 4 = elite/international, 5 = world class

### Environmental Conditions and Medical Events

Hourly environmental data (ambient air temperature, relative humidity, wind speed and solar radiation) were collected retrospectively from the closest Hong Kong Observatory [21] station (Happy Valley, 2.2 km from race venue) for 2 weeks prior to the event. The weather station was not shaded by nearby hillsides or skyscrapers throughout the race period. The WBGT was calculated using an online tool [22]. Data are interpreted against the heat stress categories of World Triathlon, encompassing five coloured flags up to a maximum allowable competition temperature of 32.2 °C WBGT [11]. World Triathlon categories are as follows: ‘*low’* (*Green* flag, < 25.7 °C WBGT); ‘*moderate’* (*Blue* flag, 25.7–27.8 °C WBGT), ‘*high’* (*Orange* flag, 27.9–30.0 °C WBGT), ‘*very high*’ (*Red* flag, 30.1–32.2 °C WBGT), or ‘*extreme’* (*Black* flag, > 32.2 °C WBGT). All medical events requiring treatment were reported from five aid stations, with anonymised medical data provided by World Triathlon. Medical event classification was completed by the medical personnel at each location, with treatment logs later provided to the Chief Medical Officer.

### Athlete Measurements

Athletes aged > 18 years old were provided with two ingestible telemetric temperature pills (e-Celcius, BodyCap, France) to measure T_CORE_. One was swallowed the night before the race and one upon waking on race day. This provided a back-up measure of T_CORE_ if the first pill was excreted. Data were downloaded post-race via a BodyCap ‘gateway’. T_TORSO_ was measured using a flexible thermistor (e-Flex, Bodycap, France), affixed to the torso at T5 level using a breathable film patch (Tegaderm, 3 M, USA) or using a mesh bandage and placed inside a heart rate chest strap (Polar, Kempele, Finland). T_CORE_ and T_TORSO_ data were logged every 30 s. Only athletes aged > 18 years (*n* = 44) were provided telemetric pills, based on manufacturer contraindications. T_TORSO_ sensors were only available for World Cup and HK-Elite athletes (*n* = 42). An inertial sensor (Runscribe, San Francisco, USA) was attached to the left shoelaces to measure running kinematics including ground contact time, stride frequency and stride length in all athletes. Foot pods were calibrated following manufacturer recommendations, including stature and mass. Race timing data were extracted from official World Cup [23] and HK-Elite/Amateur race results [24]. 

### Statistical Analysis

Data are reported as mean ± SD. Thermal and biomechanical data were analysed using two-way ANOVA (race category*time), with Bonferroni correction. Thermal data were analysed across the end of each race phase (swim, bike and run) and biomechanical data across each 1 km of the run. Differences in peak T_CORE_ and T_TORSO_ across race categories (i.e. World Cup, HK-Elite, Amateur) were investigated using one-way ANOVA, with Bonferroni correction. Peak values were included until 3 min post-race, ensuring data were captured that could relate to EHI in the finish area. Analysis was conducted using R, RStudio v.2023.12.1.402 [25] and *tidyverse* [26], with significance *p* < 0.05.

## Results

### Environmental Data and Medical Events

The race occurred in a heatwave, with the highest daily March temperature since 1884 (Fig. 1). World Triathlon published water temperatures for World Cup races were 22.1 °C (females) and 22.3 °C (males). From 538 competitors, 35 medical events were reported across all races; abrasions (*n* = 16), miscellaneous fatigue (*n* = 12), exercise-associated muscle cramps (*n* = 2), blister (*n* = 1), nausea (*n* = 1), ankle sprain (*n* = 1), dizziness (*n* = 1) and heat exhaustion (*n* = 1). None of these medical events involved participants from within the instrumented cohort.

### Thermal Responses

**Fig. 2:**
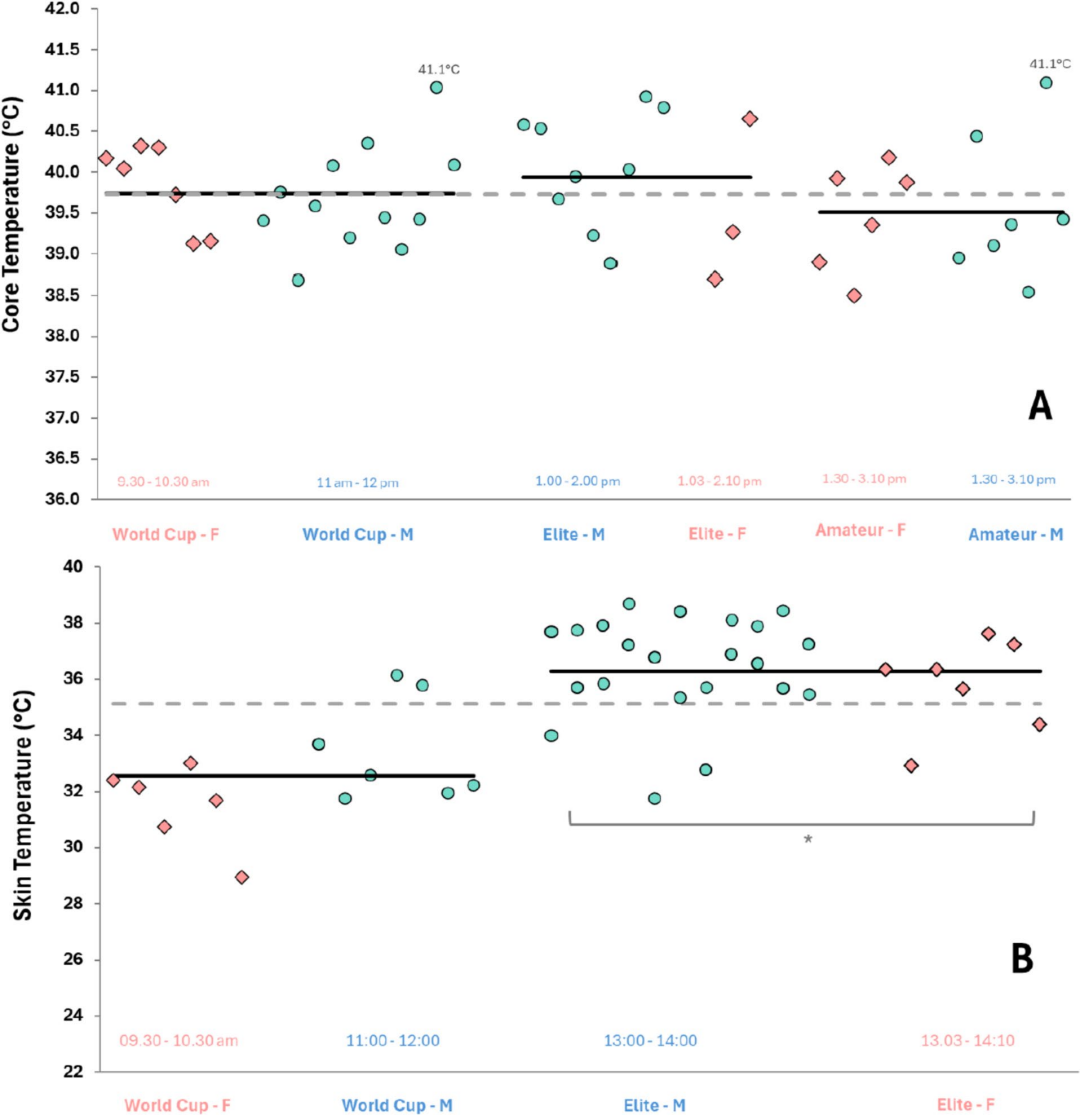
Peak core temperature (upper panel—**A**) and peak skin temperature (lower panel—**B**) presented by race category and sex. Skin temperature measured at the torso. *Red markers* denote females, *green markers* denote males. *Solid black line* represents race category group mean. *Dotted grey line* represents grand mean. * Difference between World Cup and Hong Kong-Elite athletes (*p* < 0.05)

**Fig. 3:**
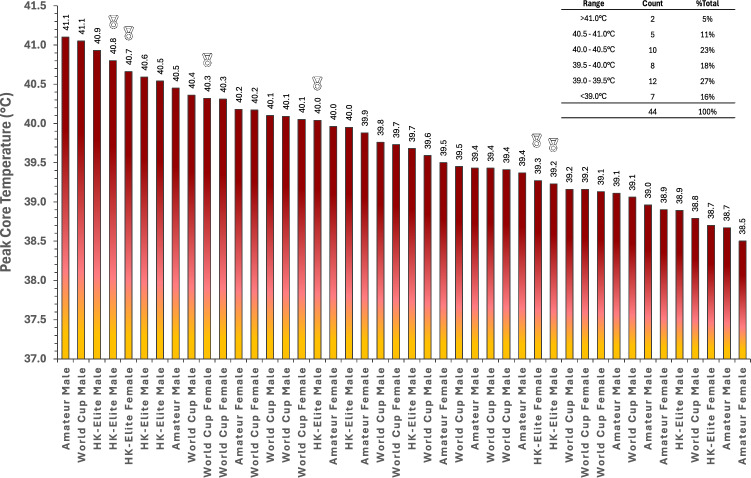
Distribution of peak core temperature during World Cup, Hong Kong-Elite (HK-Elite) and Amateur races at Hong Kong Triathlon World Cup 2024. Each column represents an individual value with medals symbolising data points of medallists in the corresponding event. Data labels are rounded to 1 decimal place. Instrumented athletes represented 18% of World Cup participants, 100% of HK-Elite participants and 3% of Amateur participants. Displayed core temperature ranges (top right) include the lower range value, but exclude the upper range value (e.g. range '39.0–39.5 °C' includes 39.0 °C but excludes 39.5 °C

Of 44 T_CORE_ records, 28 were from the second telemetry pill, with these individuals having excreted the first pill. Amateur athletes had a higher starting T_CORE_ than World Cup athletes (*t*_(146)_ = 4.16, *p* = 0.002), but not HK-Elite athletes (*t*_(146)_ = 2.97, *p*_bonf_ = 0.105, Table 2). HK-Elite and World Cup athlete’s starting T_CORE_ were not different (*t*_(146)_ = 0.97, *p* = 1.000). There was an effect of race phase (i.e. end of swim, bike and run) on T_CORE_ (*F*_(3,146)_ = 118.21, *p* < 0.001, Table 2) and an interaction between race phase and category (*F*_(6,146)_ = 3.84, *p* = 0.001). Amateur athletes displayed no differences in T_CORE_ between any sequential phases of the race (Table 2). However, T_CORE_ in both HK-Elite and World Cup athletes were higher after the bike compared with end of the swim (both *p*_bonf_ < 0.001), and after the run compared with after the bike (both *p*_bonf_ < 0.001, Table 2). Peak T_CORE_ was not different between categories (*F*_(2,41)_ = 1.06, *p* = 0.357, Fig. 2), with the distribution of peak T_CORE_ in the range 38.5–41 °C (Fig. 3).

**Table 2:** Thermal responses to an international sprint triathlon, presented by race category (World Cup, Hong Kong-Elite [HK-Elite], Amateur) and sex

Category		*n*	Race start (°C)	Swim end (°C)	Bike end (°C)	Run end (°C)	Peak (°C)	Race Δ (°C)
**T**_**CORE**_								
World cup	Female	7	36.9 (0.4)	37.7 (0.7)	38.7 (0.8)	39.7 (0.5)	39.8 (0.5)	2.5 (0.3)
	Male	12	37.1 (0.4)	37.4 (0.5)	38.7 (0.8)	39.6 (0.6)	39.7 (0.6)	2.4 (0.5)
	***Mean***	***19***	***37.0 (0.4)***^*#*^	***37.5 (0.6)***^*#,b*^	***38.7 (0.8)***^*b*^	***39.6 (0.5)***^*a,b*^	***39.7 (0.6)***	***2.4 (0.4)***
HK-Elite	Female	3	37.7 (0.6)	37.8 (0.5)	38.6 (0.9)	39.4 (0.8)	39.5 (1.0)	1.7 (1.0)
	Male	9	37.1 (0.6)	37.3 (0.4)	38.8 (0.8)	40.0 (0.7)	40.1 (0.7)	2.8 (0.8)
	***Mean***	***12***	***37.3 (0.6)***	***37.4 (0.5)***^*^,b*^	***38.8 (0.8)***^*b*^	***39.8 (0.8)***^*a,b*^	***39.9 (0.8)***	***2.5 (1.0)***
Amateur	Female	6	38.2 (0.3)	38.6 (0.4)	38.9 (0.5)	39.5 (0.6)	39.5 (0.7)	1.3 (0.5)
	Male	7	37.8 (0.2)	38.2 (0.4)	38.9 (0.4)	39.5 (0.9)	39.6 (0.9)	1.7 (0.7)
	***Mean***	***13***	***38.0 (0.3)***	***38.4 (0.4)***	***38.9 (0.4)***	***39.5 (0.7)***^*a*^	***39.5 (0.8)***	***1.5 (0.7)***
Overall		44	37.4 (0.6)	37.8 (0.7)	38.8 (0.7)	39.6 (0.7)	39.7 (0.7)	2.2 (0.8)
**T**_**TORSO**_								
World cup	Female	6	30.4 (1.6)	22.7 (1.2)	29.9 (1.1)	30.3 (1.8)	31.5 (1.5)	− 0.1 (1.2)
	Male	7	31.7 (1.5)	23.3 (1.6)	32.7 (2.3)	32.6 (1.5)	33.5 (1.8)	1.1 (0.9)
	***Mean***	***13***	***31.0 (1.6)***	***23.0 (1.4)***^*b*^	31.4 (2.3)^*,b^	31.4 (2.0)^*^	***32.6 (1.9)***	***0.5 (1.2)***
HK-Elite	Female	6	32.4 (1.6)	23.5 (0.9)	34.2 (1.1)	35.8 (1.2)	36.3 (1.2)	3.4 (2.1)
	Male	22	32.6 (2.2)	23.1 (1.0)	35.7 (1.7)	35.7 (2.1)	36.5 (1.8)	3.0 (1.5)
	***Mean***	***28***	***32.5 (2.0)***	***23.2 (0.9)***^*b*^	***35.4 (1.7)***^*b*^	***35.8 (1.9)***^*a*^	36.4 (1.7)^*^	***3.1 (1.6)***
Overall		41	32.1 (2.0)	23.2 (1.1)	34.1 (2.6)	34.5 (2.8)	35.2 (2.5)	2.4 (1.9)

Race Δ denotes difference between measurement at race start and finish. Peak = highest 30-s average during the race

*Difference between World Cup and HK-Elite

^#^Difference between World Cup and Amateur

^Difference between HK-Elite and Amateur

^a^Difference between start and end of race

^b^Difference from end of previous phase

One T_TORSO_ sensor was removed by an athlete during the warm-up or the race, leaving 41 records for analysis. There was an effect of race phase on T_TORSO_ (*F*_(3,151)_ = 371.98, *p* < 0.001, Table 2). Peak T_TORSO_ was higher in HK-Elites during afternoon races compared with World Cup athletes (*F*_(1,151)_ = 68.33, *p* < 0.001, Fig. 2). There was an interaction between race phase and category (*F*_(3,151)_ = 11.09, *p* < 0.001). HK-Elite and World Cup T_TORSO_ reduced comparably during the swim (HK-Elite: *t*_(151)_ = 19.48, *p*_bonf_ < 0.001; World Cup: *t*_(151)_ = 11.5, *p*_bonf_ < 0.001), with a greater increase then observed for HK-Elite during the bike (HK-Elite: *t*_(151)_ = − 25.93, *p*_bonf_ < 0.001; World Cup: *t*_(151)_ = − 12.24, *p*_bonf_ < 0.001), whilst neither changed during the run (HK-Elite: *t*_(151)_ = − 0.88, *p*_bonf_ = 1, World Cup: *t*_(151)_ = 0.05, *p*_bonf_ = 1.00).

### Biomechanical Responses

**Fig. 4:**
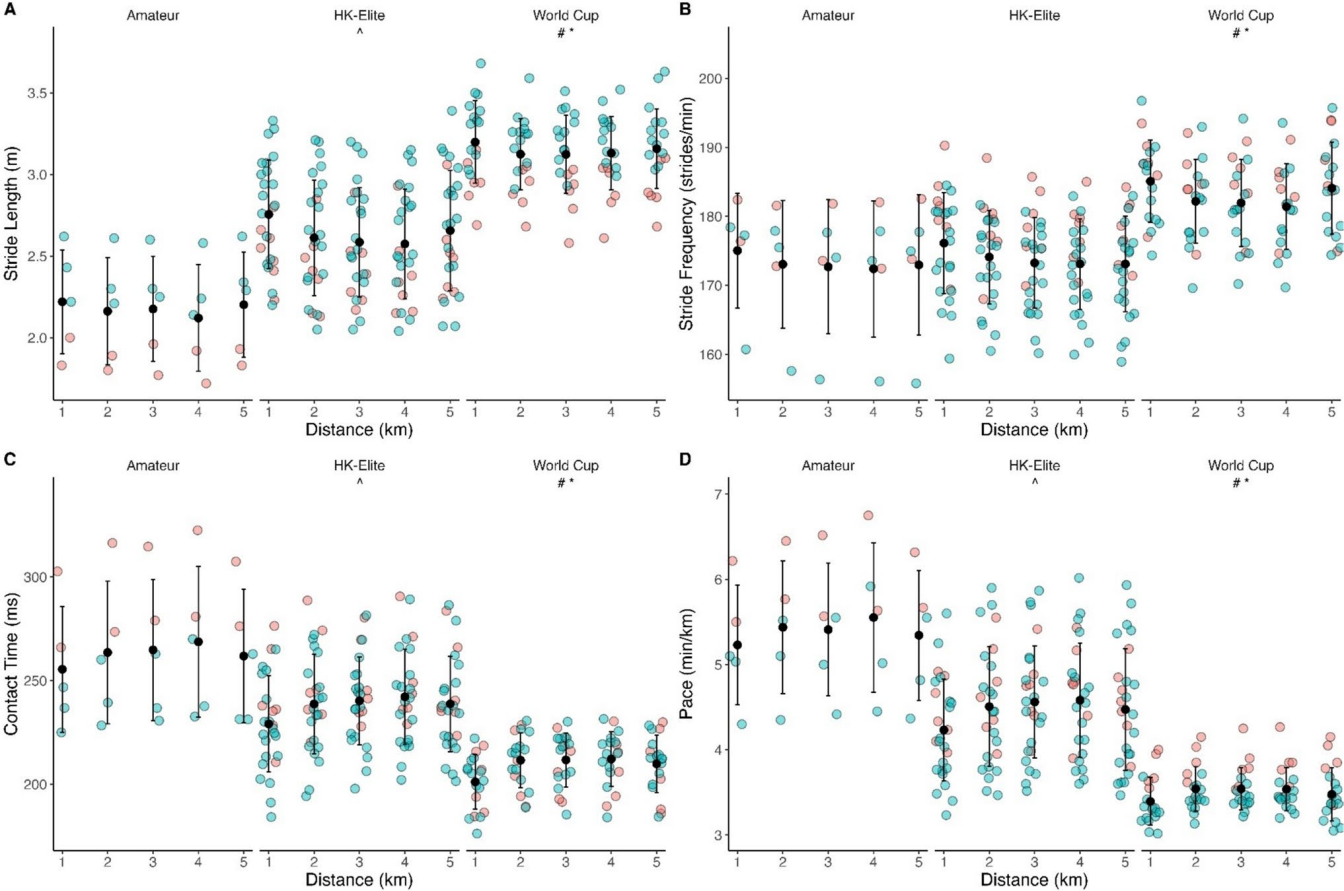
Running biomechanics during 5-km run phase of sprint triathlon, presented across each 1-km split by race categories (World Cup, Hong Kong-Elite [HK-Elite], Amateur) and sex. Panel **A** = stride length, **B** = stride frequency, **C** = contact time, **D** = pace (time per km). *Difference between World Cup and HK-Elite. ^#^Difference between World Cup and Amateur. ^Difference between HK-Elite and Amateur. *Red markers* indicates females, *green markers* indicates males. *Error bars* represent mean ± standard deviation (SD)

Sixteen records of running kinematics were unable to be downloaded (*n* = 11) or revealed corrupted data (*n* = 5). For all remaining running kinematic variables (*n* = 50), no differences were found across 1-km splits and there were no interaction effects (Fig. 4). However, pace differed across race categories (*F*_(2,244)_ = 146.56; *p* < 0.001), with World Cup athletes (03:29 ± 00:15 min.km^−1^) faster than HK-Elites (04:28 ± 00:38 min.km^−1^, *t*_(247)_ = 12.86, *p*_bonf_ < 0.001) and Amateurs (05:24 ± 00:46 min.km^−1^, *t*_(247)_ = 15.06, *p*_bonf_ < 0.001). HK-Elites were also faster than Amateurs (*t*_(247)_ = 7.56, *p*_bonf_ < 0.001). Stride length differed between categories (*F*_(2,244)_ = 132.97; *p* < 0.001). World Cup athletes (3.15 ± 0.23 m) displayed longer strides than HK-Elites (2.64 ± 0.35 m, *t*_(247)_ = − 12.47, *p*_bonf_ < 0.001) and Amateurs (2.18 ± 0.30 m, *t*_(247)_ = − 14.25, *p*_bonf_ < 0.001), which also differed (*t*_(247)_ = − 6.96, *p*_bonf_ < 0.001). Stride frequency differed across categories (*F*_(2,244)_ = 52.47; *p* < 0.001). World Cup athletes (182.9 ± 6.3 strides.min^−1^) took more strides than HK-Elites (173.9 ± 6.8 strides.min^−1^, *t*_(247)_ = − 9.75, *p*_bonf_ < 0.001) and Amateurs (173.2 ± 8.7 strides.min^−1^, *t*_(247)_ = − 6.31, *p*_bonf_ < 0.001), which did not differ (*t*_(247)_ = − 0.47, *p*_bonf_ = 0.636). Contact time was different across categories (*F*_(2,244)_ = 86.34, *p* < 0.001), with World Cup athletes (209.3 ± 13.7 ms) shorter than HK-Elites (237.8 ± 23.0 ms, *t*_(247)_ = 10.06, *p*_bonf_ < 0.001) and Amateurs (262.9 ± 31.0 ms, *t*_(247)_ = 11.35, *p*_bonf_ < 0.001). Contact times of HK-Elites were also shorter than Amateurs (*t*_(247)_ = 5.46, *p*_bonf_ < 0.001).

## Discussion

We document the thermal and biomechanical responses to an early-season international sprint triathlon, occurring during unseasonably hot weather, which eclipsed record daily peak temperatures for March since 1884. From 44 athletes monitored for T_CORE_, 17 displayed a T_CORE_ > 40 °C (39%). World Cup, HK-Elite and Amateur athletes presented comparable peak thermal responses, with no apparent differences in presentation of T_CORE_ > 40 °C between amateur and higher trained athletes. Four of seven World Cup females presented with T_CORE_ around 40 °C, despite this race occurring under *Green/Blue* flag conditions (i.e. ‘low-moderate’ heat stress). Peak T_CORE_ demonstrated considerable inter-individual variability (range 38.5–41.1 °C, Fig. 3), which reinforces recent calls to explore multi-parameter monitoring of individuals as part of a strategy for reducing EHI incidence, beyond tracking environmental heat stress and/or T_CORE_ alone. The magnitude of thermal strain observed (i.e. 2 athletes > 41 °C) during a sprint distance triathlon questions current safety guidelines that suggest reducing race distance as a preventative strategy against EHI when environmental conditions are more severe, such as *Red/Black* flag scenarios, given an intuitive greater risk of EHI with elevated T_CORE_.

### Unpredictable Weather

A heatwave was experienced at this event, with race-day conditions ~ 4 °C WBGT above the prevailing weekly average (Fig. 1). With climate change, such extreme weather patterns are expected to increase over the next decade [27]. The risk of EHI during sprint triathlons appears greatest during events early in the season (typically March–September [28]), when athletes likely have minimal natural acclimatisation [29]. As little as 4 days of training in a hot environment may confer some protective benefits [30], but integrating heat acclimation into triathlete’s demanding training schedules more likely requires two training weeks for comprehensive adaptations [31]. Nevertheless, the rate of temperature change (Fig. 1) likely eliminated any protective acclimatisation effect for most international athletes arriving in Hong Kong 4–5 days prior to race day [32]. On race day, *Green/Blue* flag conditions were expected, but by midday were *Red*, continuing thereafter (Fig. 1). Thus, lower standards of athletes (i.e. HK-Elite and Amateur) competed in more severe environmental conditions than World Cup athletes. Whilst no differences in peak T_CORE_ were found between categories, peak T_TORSO_ was higher in HK-Elites (afternoon) than World Cup athletes (morning), indicating a degree of increased physiological strain due to the conditions (Fig. 2). Despite this, only one medical event (from 538 race entrants) was classified as heat exhaustion across the competition. However ‘*miscellaneous fatigue*’, ‘*muscle cramps*’, ‘*dizziness*’ and ‘*nausea’* could present during the initial stages of EHI [33], of which there were 15 occurrences. These individuals were potentially also suffering from heat exhaustion given environmental conditions on the day. Environmental temperature remains a leading cause of illness in short-course triathlons [19]. Whilst early-season races represent the period of greatest risk, it should be noted that in summer months, absolute temperatures are higher, thereby further challenging thermoregulation. The extent to which amateur triathletes will accrue seasonal acclimatisation sufficiently to support sustained higher intensity exercise is unclear. Coupled with recent proposals advocating more conservative sport-specific heat stress guidelines in sports including triathlon [34], accurately assessing risk based upon environmental conditions remains a challenging area [10].

Our data indicate recently proposed sport-specific thresholds [34] may be too conservative, given these proposals were modelled against a T_CORE_ threshold of > 40.0 °C and the prevalence with which we, and others, report athletes routinely exceeding this [4–9]. Race organisers and International Sporting Federations (ITF) may therefore benefit from partnering with meteorological centres to ensure they receive the latest weather forecasts and regularly update both organisers and participants in the days and weeks prior to events. This can enable the prompt implementation of relevant acute and chronic strategies for health and performance from both athlete and organiser perspectives [35].

### Physiological Responses

Peak T_CORE_ was similar across race categories (39.7–39.9 °C) and consistently observed at the end of the race (Table 2). The change in T_CORE_ was greater in HK-Elite and World Cup athletes, which likely reflects greater exercise intensity and associated metabolic heat production and/or lower body mass in higher trained individuals. Consistent changes in T_CORE_ for HK-Elite and World Cup athletes across each phase of the race indicates uncompensable heat stress for these categories. The current data also include some of the few observations of amateur athletes (predominantly Tier 1) presenting with peak T_CORE_ > 40 °C (*n* = 3/13), which included one case > 41 °C. This reinforces the need for further monitoring of individuals during real competitions, as ethical regulations preclude these perspectives within laboratory trials [36]. A recent review of sporting T_CORE_ responses reported no cases of amateur athletes with T_CORE_ > 40 °C [18]. Singh et al. reported ~ 12% of elite athletes presenting with T_CORE_ between 40 and 41.5 °C during competition, of whom ~ 3% reported EHI symptoms [18]. This compares with 39% of our cohort between 40 and 41.5 °C, with zero EHI. This indicates amateur athletes can tolerate T_CORE_ > 40 °C without symptoms of EHI and reinforces the limitations of T_CORE_ alone as a predictor of EHI [1, 37].

To our knowledge, these are the first competition data from a sprint distance triathlon including elite athletes and demonstrate comparable thermal strain to the limited data pertaining to Olympic distance events (i.e. 1.5-km swim, 40-km bike and 10-km run). Peak T_CORE_ between 38.8 and 39.7 °C were reported from five athletes competing in conditions of ~ 19 °C and 55% relative humidity [38]. Unlike half and full Ironman triathlons, we did not observe a plateau or reduction in T_CORE_ after the swim [39, 40], indicating uncompensable heat stress during sprint distance races, likely a consequence of greater exercise intensity and metabolic heat production during the shorter distance event [41]. Despite the absence of heat illness incidence within the current cohort, the presentation of comparable or even more severe thermal responses during a sprint distance event calls into question the suitability of reducing race distance as a preventative strategy against EHI under more severe heat stress.

There were comparable peak T_CORE_ responses between HK-Elite and World Cup athletes, however T_TORSO_ was ~ 3–4 °C higher in the afternoon HK-Elite race (~ 5 °C WBGT hotter). Monitoring skin temperature alongside T_CORE_ is recommended to assess overall thermal state [6]. The similarity of T_CORE_ responses between race categories in this event support thermal monitoring beyond T_CORE_ alone, given that World Cup athletes demonstrated faster bike and run performances, whilst racing in cooler conditions. Skin temperature has a large influence on both cardiovascular and perceptual strain during endurance competition [42] and heat exchange with the environment can be better understood by the gradient between T_CORE_ and the skin. Pragmatic challenges with skin temperature measurements during competition remain however, especially with multi-site measurements preferred, given known inter-limb skin temperature differences [43]. However, multiple skin temperature sensors present potential comfort concerns for athletes, which limited measurement to a single torso sensor in the current study. Non-contact measurements such as thermal imagery offer convenience for measuring multiple athletes and large areas of skin [4], but accuracy is compromised by factors such as sweat/water on the skin and measurement distance and angle [44]. Furthermore, the infrastructure is currently lacking to deploy across multiple measurement locations during a competition and receive live synchronised data to identify athletes at risk of EHI. Notwithstanding these challenges, T_TORSO_ revealed a marked effect of water immersion during swimming (~ − 9 °C T_TORSO_), a magnitude comparable to ‘external’ precooling strategies which for a limited time afford lower HR and perceived exertion [45]. A ~ 1 °C increase in T_CORE_ occurred during the bike phase, despite considerably lower T_TORSO_ and factors supporting more efficient thermoregulation such as air flow during cycling, wet skin and clothing. This highlights risks associated with events with warmer water temperature [46] or no swimming (e.g. duathlon), where these factors may not support thermoregulation as effectively.

### Sex Differences

Our sample sizes precluded a robust comparison of sex responses across race categories. However, no clear sex differences were evident across peak or change in body temperatures (Fig. 2 and Table 2). Historically, females are purported to have a thermoregulatory disadvantage, due to a maximal lower sweat rate [47]. However, recent evidence indicates sex did not independently influence changes in body temperature [48]. Accordingly, our data support the conclusion that males and females do not require different regulations for endurance events in hot environments [48].

### Running Biomechanics

We found differences in pace, stride length, stride frequency and contact time between all race categories, apart from HK-Elite and Amateur stride frequency. However, no changes were found across the 5-km run, with no visible pacing strategy or apparent fatigue effect. Whilst biomechanical variables may be able to characterise EHI in severe circumstances [12], there appears no clear relationship between core temperature and changes in endurance running biomechanics [49]. Future research and monitoring approaches may consider analysing smaller intervals, as 1-km averages may have included too much data to detect modest changes.

### Limitations

We acknowledge the potential for fluid ingestion to alter some of the readings, given 28 records were derived from the second pill, with the first pill already excreted. Some T_CORE_ responses may therefore have been *higher* than we report. The use of telemetry pills remains financially and logistically challenging when working with individuals who compete in the morning. Using telemetric capsules by rectal insertion may be one approach to negate this ingestion period limitation [50], although pilot testing in athletic scenarios is necessary and familiarisation with this technique may be required. We prioritised data collection from World Cup and HK-Elite athletes. Therefore, we monitored fewer amateur participants (*n* = 13), which may introduce sampling bias given this was the largest participation category.

### Research and Policy Implications

Despite minimal EHI cases, severe thermal responses (2 individuals > 41 °C) occurred during a sprint triathlon, which is a stated modification purported to lower risk for Olympic-distance triathlon. Therefore, reducing triathlon events to a ‘sprint’ distance may not sufficiently reduce the risk of heat illness, utilising earlier start times may be a more suitable strategy. Further collection of physiological data from varied athlete cohorts during sprint events held under *Red/Black* conditions (maximum permissible heat stress) are needed to develop heat policies specifically for triathlon.

Environmental data offers limited insights into EHI risk, given the individual differences observed. Notwithstanding, given the temperature variability around this event, ITFs should consider partnering with meteorological institutions to predict event weather as accurately as possible and communicate this regularly to competitors in advance. A variety of passive [51], active and passive [31], or acute heat alleviation strategies [52] can aid performance and health of athletes even at short notice.

Event organisers should consider the order of races. In this event, athletes of lower training status competed in the hottest conditions. Amateur athletes often represent the largest participation category. Despite comparable T_CORE_ responses, there remain other pertinent physiological differences (sweat rate, blood volume, perceptual tolerance), that may put lower trained individuals at greater EHI risk [53].

We advocate for continued development of robust and ergonomic sensors that can withstand multi-mode exercise events, especially water immersion, whilst providing real-time data. Multi-sensor monitoring, beyond T_CORE_ alone, is recommended to better appraise the risk of EHI. Further normative data are needed to develop robust and sport-specific EHI policies that cater for a large range of individuals and locations, different race distances and times of the year. Such policies should consider real-time monitoring, given the variable heat strain observed and unpredictable weather patterns which are likely to increase in frequency.

### Conclusion

The presentation of core temperatures > 40 °C appears to be independent of athlete level. Core temperature may rise > 41 °C during a sprint triathlon held under *Green/Blue* flag conditions, questioning whether this remains a suitable EHI safety mitigation strategy for Olympic-distance triathlons held under *Red/Black* flag conditions (> 30.1 °C WBGT).

## Declarations

### Data Availability

The data are not available to the public, as they include those of professional athletes. Authors are willing to share code upon request.
